# Neuronal function is necessary but not sufficient for consciousness: consciousness is necessary for will

**DOI:** 10.3389/fnint.2012.00103

**Published:** 2012-11-20

**Authors:** David Nussbaum, Khadija Ibrahim

**Affiliations:** ^1^Department of Psychology, University of Toronto ScarboroughToronto, ON, Canada; ^2^Forensic Program, Ontario Shores for Mental Health SciencesWhitby, ON, Canada

**Keywords:** will, consciousness, information representations, necessary not sufficient, neural representations of information

## Abstract

Behavioral neuroscience has presented philosophers with the task of clarifying the relationship between neural determinism and free will. If neural functions encode information and govern decision-making, are the constructs of will, agency and indeed morality illusions of pre-scientific ignorance? This article will argue that neuronal function is necessary for representing distinct sensory-perceptual, cognitive, motivational, emotional states, and motor functions. However, neural transmission and action potentials are simply chemical-physical representations of these informational states but are not the embodiment of consciousness itself. By some yet undiscovered mechanism, consciousness “reads” the neuronal events into conscious experience. Absent a particular specialized brain region or sufficient relevant transmitters and receptors, relevant information cannot be processed and the individual cannot be conscious of that informational state. In natural and many artificial communication systems, communications proceed bi-directionally. By an argument of symmetry, if neuronal activity can communicate with consciousness, there is no reason to preclude consciousness from communicating back and influencing neuronal function. In the intervening conscious moment, information from diverse perceptual, motivational, cognitive, and emotional sources is weighted and will results. This process then biases resultant neural processes to actualize the willed target. This approach is limited in terms of operationalization into an experimental study because at present, there is no method to measure consciousness-independent of neuronal function and subjective report.

The notion of will has engaged religious thinkers, philosophers, and secular moralists since ancient times. More recently neuroscientists have entered the debate with ongoing advances in untangling the workings of the brain to explain different aspects of behavioral outcomes. Implications for moral and legal systems are profound. With amorality at one end and divine evaluation of every human deed at the other, free-will is what humans use to base their accounts and judgments of others. Neuroscientist Sperry (1976a) discussed two issues, *conscious awareness* and *free-will* almost four decades ago but their parameters remain unresolved through the intervening period of scientific inquiry and advancement. It is our position that the two are intertwined to produce behavior via brain mechanisms that generate subjective conscious and evaluative experiences that under appropriate conditions, afford responding to stimuli in ways that we desire. From our perspective, the ability to will actions appears to rest on consciousness.

Behaviorists asserted that all meaningful behavior was determined by stimulus response laws, leaving no room for will. With the advent of behavioral neuroscience, researchers adduced considerable evidence that specific behaviors have anatomical and neurotransmitter/receptor “homes” and have argued that since information processing follows identified, regular patterns, behavior is determined by the neural chemical-physiological mechanisms rendering behavior causally determined by neural information processing (Sperry, 1976b), essentially precluding will and moral decision-making. This paper will present what we perceive as a lacuna in neurobiological accounts of consciousness and as a consequence, a possible alternative that definitely accepts that neurobiological processes are a necessary, but insufficient condition for consciousness. Consciousness in our perspective is the lynchpin between neurobiology and will.

Neurobiological approaches typically propose neuroanatomical connections deemed necessary for consciousness to occur. To illustrate, we first provide a non-exhaustive but representative survey of the neurobiology of consciousness and then present what we see as problematic with this perspective. We argue that neurobiology is necessary for consciousness to occur with distinct neural states serving as representatives of particular informational states, but why this does not constitute consciousness itself. We propose an alternative that consciousness is intermediary between neural representations of informational states and will. By an argument of symmetry, we suggest that if neurobiology can communicate with consciousness, there is no intrinsic reason to preclude consciousness from communicating with neurobiology. It is in the reverse flow of information from consciousness to neurobiology that will is expressed.

## Definitions

We will define a few central terms to frame the discussion to follow. Consciousness has a variety of meanings in different contexts including arousal in the sleep-wakefulness continuum, self-awareness in the introspective context, qualia in terms of sensory experiences, and an inclusive general awareness of being cognizant of place, time, person, and an abstract, symbolic ability to represent the experiential perceptual, emotional, motivational, cognitive, and motor states being processed on a moment to moment basis. This more encompassing but specific conceptualization (i.e., does not directly refer to the sleep-wakefulness dimension nor does it refer to comatose patients) is related to attention (Watt and Pincus, 2004).

From an existential perspective, the question of qualia with respect to conscious experience remains despite the lack of a compelling explanation for why neurobiological processes produce rich and holistic experiences (Crick and Koch, 2003). For example, there is something “it-is-like” to experience a patch of red, to experience a sensation (i.e., pain), and these phenomenally unmistakable and deeply subjective aspects of conscious experience have been termed qualia (Crick and Koch, 2003). How we experience the redness of red stems from brain activity and the difficulty in describing one's subjective percept of such experience is still an unresolved issue. A principle goal in sensory-perceptual research focuses on isolating one-to-one relationships between neural activities and specific experiences. This approach has met with considerable success. For example, neurobiological correlates of the visual perception of shape, size, color, texture, or movement of an object are reasonably well and fully established (Crick and Koch, 2003). However, it might be argued that activity in the visual modality (and by extension, other sensory modalities) despite one's consciousness of them lies beyond will to significantly change. Research pertaining to sleep-wakefulness cycles deals with the factors needed for all forms of consciousness in the ascending reticular systems in the brainstem. Here again, researchers are presented with difficult questions of an empirical nature. However, they are strictly dependent on the neuroanatomical functions related to the unconscious state of rest during sleep. For the purposes of this paper, it is attentional processes that are involved in the production of conscious awareness and the aftermath in free-will that will ultimately set the stage for new questions on human morality and decision-making.

The five general information processing domains noted above [perceptual, motivational, emotional, cognitive (including language), and motor] contribute to consciousness to greater or lesser degrees reflecting salience-based attention in varying contexts and environments (Gallace and Spence, 2008). Thus, we will avoid overly particular (i.e., sensory-specific) notions of consciousness. Additionally, this paper is not about what ultimately constitutes morality and ethics but human ability to consciously consider the moral or ethical components of a situation and reference the situational elements against one's moral or ethical principles. It is recognized that different societies recognize different action as moral and immoral (e.g., gay pride parades are not equivalently regarded in different parts of the world). Even within specific legal systems, different players may have different and mutually exclusive ethical imperatives where the role of the practitioner is an accepted discriminant of ethical behavior. For example, mental health professionals performing forensic assessments are enjoined by professional ethical standards to provide complete and objective opinions with respect to a variety of legal issues including criminal responsibility/insanity. Lawyers' primary ethic is the best interests of their clients and not objective truth within an adversarial legal system.

## A focused review of the literature

Philosophies of mind debates have historically revolved around consciousness because of its obvious implications for agent free will, action, and ethical behavior. More recently, this has paved the way for exploring potentially inviolate relationships between neurobiological states and behavioral outcomes. The overarching question relates to whether consciousness of voluntary actions is experienced without sufficient neuropsychological determinative causal conditions or states (i.e., that they are free; Searle, 2000). Philosophical researchers have aimed to unravel consciousness by conducting empirical studies. However, the deductive approach has proved inadequate in addressing the question of where consciousness is and how it operates. The general definition that was used to describe consciousness earlier in the field of psychology was “primary” level consciousness which is “an awareness of one's surroundings, of the self, and of one's thoughts and feelings” (Sommerhoff and MacDorman, 1994). Sommerhoff and MacDorman (1994) distinguish primary level consciousness from other abstract levels that are derived from language and propositional knowledge. The distinction in levels psychologically is theoretical and perhaps arbitrary for the purpose of anatomical and empirical research when focusing on a type consciousness in the five processing domains of attention. Turner and Knapp (1995) used Sommerhoff and MacDorman's theoretical framework of consciousness to draw out evidence of primary consciousness in cerebral structures by inducing electrical stimulation to produce an experience that is directly apprehended in human subjects. The occipital, parietal and frontal lobes produced experiential responses, while Broca's and Wernicke's speech areas produced speech upon electrical stimulation (Turner and Knapp, 1995). The argument made by these scholars was that only the structures of the anterior temporal lobe can afford evoked experiences of consciousness. That is, upon electrical stimulation of the anterior temporal lobe structures including the temporal cortex, hippocampus and amygdala, realistic visual and auditory experiences were evoked. However, they added that the temporal lobe does not act independently but rather is part of an anatomical system of relays with other brain regions (Turner and Knapp, 1995).

More recent efforts attempted to refine these conclusions by arguing that although anatomically specific brain regions are necessary for the experience of consciousness, they are not sufficient. For example, Baars and Laureys (2005) specifically propose that the philosophical effort of Block to include cognitive neuroscience lacks grounding due to the limited nature of Block (2005) interpretation of cognitive neuroscience (Baars and Laureys, 2005). The two types of consciousness that Block (2005) presents are “phenomenological consciousness” (what we experience) and “access consciousness” (the information we can access via conscious experience) which Baars and Laureys contend are necessary but not sufficient for conscious qualities. The point made is that the contents of visual consciousness for example, require the visual cortex but activity in the visual cortex is not sufficient for conscious qualities (Baars and Laureys, 2005). Furthermore, researchers have found no correlation between visual cortices and consciousness without parietal and prefrontal activation (Dehaene et al., 2001). Baars and Laureys make it a point to state that *unconscious* brain mechanisms are necessary for conscious experience but reject Block's (2005) notion of “access consciousness” because stimulation within the brain does not evoke conscious experience, while they remain necessary for the experience of consciousness itself (Baars and Laureys, 2005).

Searle (2000) raised the question of whether will is a subsumed feature of neuroanatomy. However, Searle situates his discussion on the consciousness of free action within an account of anatomical structures of the brain. The notion that consciousness can be reduced to conscious behavior or to functional states of a system, is replaced by the more commonly accepted belief that consciousness is indeed a biological phenomenon which consists of sentience, awareness, thoughts, and feelings (Searle, 2000). Klemm (2011) sees consciousness more anatomically grounded. His “Conscious System” involves active circuitry between the brainstem reticulum, basal forebrain, thalamus, and neocortex with the brainstem ascending reticular arousal system (ARAS) activating areas of neocortex to generate consciousness and wakefulness (Klemm, 2011). This position invokes features of consciousness resulting from activation of specific aspects of neuroanatomical activation.

More restricted examples of sensory-level analyses of consciousness have been made for the olfactory system Keller (2011). While arguably necessary for specific sensory experiences, these focal experiences do not capture the multiplicity of consciousness in a holistic manner and may be irrelevant to the direct neurobiological dynamics of will. It is the summation of all those and more that produces consciousness and subsequently, will of action is the translation of “selection of stimuli and choice of response” as first mentioned by James (1980/1950) and (Ingvar, 1994). Discussions on the role of attention in consciousness awareness have been an important part of the empirical research conducted on consciousness due to the fact that attention can be varied experimentally which in turn affect conscious awareness (Keller, 2011). Ingvar (1994) argued that conscious awareness is a prerequisite for willful acts, since willful acts include active (i.e., directed) attentional mechanisms.

As an example of a specific sensory information processing system, the neural pathways deemed necessary for visual perception encompassing consciousness is characterized by complimentary streams leaving the occipitally located primary visual cortex. The dorsal stream projects to the parietal lobe, then the frontal lobe, and then both project to the corpus striatum (McIntosh et al., 1994). The ventral stream projects to the cortex of the anterior temporal lobe to the amygdala and hippocampus. The dorsal stream situates the visual array within three-dimensional space enabling guided motor behavior (Taylor, 1997). Alternately, the ventral stream affords the ability to identify, remember, and recognize the heuristic and emotional significances of objects of perception (McIntosh et al., 1994). Turner and Knapp (1995) also note that although the visual modality has perhaps been the most thoroughly researched, sensory information from other sensory modalities also relay into complex, meaningful forms that generate conscious awareness of the stimuli that activate particular sense organs, activate modality specific pathways and are experienced as distinct properties with unique sensory qualities. Turner and Knapp (1995) conclude their argument that consciousness is localized in the temporal lobe by showing that with bilateral anterior temporal lobe leucotomy in humans, there are resultant and severe deficits in object discrimination, emotional responding and inability to recognize faces. We note that it is only these informational specifics that are precluded from consciousness; not consciousness *per se* as illustration by retention of consciousness of other informational states.

Baars and Laureys (2005) contend that one cannot equate prerequisites for consciousness with the source of consciousness. An activated temporal lobe may be required for human conscious experience of some informational states. However, they themselves are not equivalent to conscious experience *per se*. We take this as our point of departure from notions of neuroanatomically situated models for consciousness that appear intrinsic to positions advanced by Searle (2000), Klemm (2011), Ingvar (1994), Keller (2011), and Ungerleider and Haxby (1994), although we clearly endorse the necessary neurobiological activation of distinct information processing networks for expressing particular types of sensory/perceptual, cognitive, motivational, emotional, and motor information.

## Proposed alternative approach

We propose the following thought experiment to illustrate our initial difficulty with neuroanatomically embedded models of consciousness. Let us assume a candidate “Network of Consciousness (NoC)” that involves a Hebbian circuit with nodes involving the Brain Stem Reticular System (affording sufficient arousal), the basal forebrain, thalamus and neocortex similar to that proposed by Klemm (2011) above. Let us further assume that one was able to slice 50 mm of rat brain in such a fashion as to dissect out connected neurons from these four anatomical sites. The 50 mm slice was then put onto a Petrie dish filled with physiological saline solution. Stimulating and recording electrodes were placed in the first and third neurons within this circuit and repeated stimulations of the initial neurons caused numerous circuit volleys of electrical activity clearly measured by the recording electrode. Despite the requisite neuronal conditions being met for consciousness, it would be a stretch to consider consciousness existing in the Petrie dish. First, the neuronal events consist of potassium, calcium, and chloride ions traversing the semi-permeable axonal membranes and neurotransmitter molecules navigating the synaptic cleft, docking in appropriate receptor molecules, and releasing to await the next cycle. It is the frequency with which this cycle repeats that conveys particular informational codes that one can be conscious of. These frequency codes represent specific informational states within modality specific and non-specific (association) tracts in the brain. One could arrange an array of peanuts, raisins and feathers on two sides of a leather strap to convey particular informational states. Two peanuts, one raisin, and three feathers to the left could represent horse, two peanuts, one raisin, and three feathers to the right of the strap could represent a cow and two peanuts, two raisins, and three feathers to the left could represent a camel, and so on. The informational code is not consciousness *per se* but some informational representation is necessary to be conscious of. Unlike the famous Seinfeld episode, one cannot be conscious of nothing.^1^ Consequently, the brain has two complimentary meta-strategies for representing information. The first is the frequency code alluded to above. The second is the place code. Identical frequency trains for example, in visual and auditory cortices give rise to experientially distinct sensations. Another problem with “locating” consciousness within a particular biological object involves where in that object does consciousness arise? Considering the three basic neuronal components how might one attempt to localize or “bits of consciousness” or fractionate consciousness itself within the post-synaptic, axonal, or pre-synaptic regions?

A second major but less “localizable” aspect of consciousness is its integrating function. To use the visual example, distinct networks within the visual system represent and process five individual parameters of visual experience; shape, size, color, texture, and state of motion. The majority of mankind fortunately possesses intact brains and consciously experiences shape, size, color, texture, and motion of an object as an integrated whole; not as a mosaic of these different parameters linked together. Somehow we experience visual scenes and the objects within them as unified visual objects with all five characteristics seamlessly integrated. It is hard to explain how anatomically separated tracts cooperatively fuse different elements into a unified whole when other interacting networks that may be turned on and off simultaneously retain distinct sensory qualities. However, absent information representation within a specific tract, the individual cannot become conscious of the information that would have been represented there. At a very gross level, someone with a severed optic nerve cannot be conscious of the information that is no longer represented through that eye. More subtly, the non-intuitive phenomenon of “blind-sight” occurs when the ventral tract is disrupted, resulting in people reporting not seeing objects presented in their visual fields while producing occipital evoked potentials. Although identification is impossible, they paradoxically demonstrate the ability to catch or avoid objects thrown at them because they can still “see” the motion through their unimpaired dorsal visual stream while not reporting conscious identification of the moving object.

We proposed that “consciousness” requires intact neural information processing to exist but neural processing *per se* is not consciousness. We observe that in a sense, science is a bi-directional process with definitions and deducible explanations resulting from this “downward” (reductionist) process (e.g., Newton's Second Law: F = MA; A = ^S/^T; S = ^D/^T with M, D, and T being fundamental and measureable qualities of the Newtonian world). Non-deducible phenomena resulting from complexity (i.e., interactions of large numbers of elements within a system) are commonly accounted for by the term “emergent properties.” There are two forms of “emergent properties.” The first occurs when a basic understanding exists for why the non-deducible property emerges. For example, reactions in inorganic chemistry involve electron sharing in outer shells with consistent and predictable regularities. Organic chemistry is replete with unpredictable reactions that need to be memorized due to the inapplicability of electron sharing rules. This is explained by the extensive length of organic molecules (i.e., carbon chains) that fold over on themselves and block available electron sites from reacting with each other. We will refer to this as a “strong emergent property” because we understand why this complexity necessitates the vitiation of simpler regularities. “Weak emergent properties” occur when an inexplicable phenomenon occurs from complexity without any sense of why. Since we have shown why we argue that consciousness is not ostensibly reducible to neuronal functioning, the notion of it being an “emergent property” of neuronal complexity is essentially a meaningless phrase naming the issue requiring an explanation rather than a helpful explanation. What then is the relationship between neurobiological function and consciousness?

We propose that consciousness entails a “reader” of neural events. We do not pretend that we know what form or location or type of energy is produced through reading neural events. What we do “know” is that consciousness is a different dimension from the myriad and intricate but knowable biochemical process that are necessary to generate consciousness. To illustrate, should a pin goes through the skin of our left thumb, we do not get a digital message that a sharp, hard object has broken our skin and activated nociceptors at location *x, y, z*, but, we experience OUCH and we know where! Somehow, consciousness integrates the different parameters into a single, coherent, integrated experience.

## Consciousness and will

To this point we have argued that neurobiological processes function as representing conveying and processing informational states. These processes necessarily exist if we are to be conscious of the specific information they potentially convey. We will now argue that the concept of will can be introduced into this system. Will can result if we accept that natural communication systems are bi-directional. For example, if Mr. Songbird can chirp his desires to Mrs. Songbird, Mrs. Songbird can chirp back her shopping list. Similarly, if neurobiology can communicate in a feed-forward fashion with consciousness, by an argument of symmetry, there is no a priori reason to exclude consciousness from communicating back to neurobiology. Indeed, the principle behind neurofeedback is that one can use their “will” to alter quantitative EEG (QEEG) patterns that have been shown to be associated with some form of psychopathology or another. Passively watching brain wave patterns does not result in desired changes. Neurofeedback however has been shown to be successful in a number of areas subsequent to altering brain wave patterns. Neurofeedback (and likely biofeedback in general) would appear at least on the surface to provide empirical evidence that thoughts and desire (will) can change neuronal patterns in the retro-example of neuronal patterns being expressed as behaviors.

## Limitations and contributions

We make no claims to having an understanding of the “emergence” of consciousness from neuronal activity. We will resist the tendency for some to say that X is an “emergent property” of Y as if the phenomenon has been “explained.” Most frequently, “Emergent Property” is pulled from the hat when the individual has no clue as to how the property emerges. We make no such claims. We acknowledge than many of our neuroscience colleagues will criticize this work on the basis that in a sense, it is “unscientific” because it proposes an intrinsic limit to what scientists might 1 day comprehend. After all, if we do not know where to look or what form of energy consciousness takes, how can we build a “consciousness detector”-independent of the electrical or magnetic currents generated by or secondary to axonal transmission or chemical trails of synaptic processes? Our major goal has been to convey that although well-defined neural substrates provide informational representations necessary for consciousness, they do not embody consciousness. Rather, in some currently unidentified fashion, consciousness is a “reader” of neural informational states that produces consciousness. Will then become possible as the “reverse-engineered” information processing, running from the conscious to the neural. While not presenting a working mechanism for consciousness and will, we do produce a way of thinking about will that respects the integrity of the levels of organization, does not engage in descriptive circularity, and specifies paths for future thinking. We do not apologize for raising the possibility that there indeed may be limits to what we as humans might ultimately comprehend. However, we welcome those in opposition to address the conceptual issues that we raise.

### Conflict of interest statement

The authors declare that the research was conducted in the absence of any commercial or financial relationships that could be construed as a potential conflict of interest.

## Footnotes

^1^For our purposes, we see information in its most general sense as a reduction in uncertainty. Consequently, absent information, any possibility or possible relationship is equally probable. Determinism results from 1 to 1 correspondences resulting in no subsequent degrees of freedom.
